# Prognostic Relevance of Ischemic Late Gadolinium Enhancement in Apparently Healthy Endurance Athletes: A Follow-up Study Over 5 years

**DOI:** 10.1186/s40798-024-00680-1

**Published:** 2024-01-29

**Authors:** Gunnar K. Lund, Sharon Leptin, Haissam Ragab, Martin R. Sinn, Alexander Fierenz, Ersin Cavus, Kai Muellerleile, Hang Chen, Jennifer Erley, Phillip Harms, Anna Kisters, Jitka Starekova, Gerhard Adam, Enver Tahir

**Affiliations:** 1 Department of Diagnostic and Interventional Radiology and Nuclear Medicine, University Medical Center Hamburg-Eppendorf, Martinistr. 52, 20246 Hamburg, Germany; 2 Institution for Medical Biometry and Epidemiology, University Hospital Hamburg Eppendorf, Hamburg, Germany; 3 Department of General and Interventional Cardiology, University Heart Center, Hamburg, Germany; 4 Department of Radiology, University of Wisconsin, Madison, WI USA

## Abstract

**Background:**

In many cardiac diseases, myocardial scar tissue detected by late gadolinium enhancement (LGE) is a risk factor for cardiac arrhythmia and sudden cardiac death. Previous studies in athletes reported an increased risk for cardiac events in this group of ostensibly healthy subjects. However, the currently available longitudinal studies on this topic included fairly old marathon runners with a mean age of 57 ± 6 years or represent a case–control study in athletes with preexisting ventricular arrhythmia. The purpose of this prospective study was to analyze the prognostic relevance of LGE cardiac magnetic resonance (CMR) in middle-aged endurance athletes without known preexisting cardiac disorders.

**Methods:**

Three-hundred and twelve apparently healthy athletes were prospectively enrolled. Inclusion criteria were a training for a minimum of 10 h per week and regularly participation in competitions. LGE CMR was obtained at baseline in all athletes and presence of LGE was classified visually according to established criteria as ischemic LGE, major or minor non-ischemic LGE or absent LGE. Follow-up consisted of a standardized questionnaire and an additional phone call in case of incomplete data. An event was defined as fatal myocardial infarction, ventricular tachycardia, ventricular fibrillation or sudden cardiac death (SCD).

**Results:**

Complete follow-up was available for 293/312 athletes (94%) including 145 triathletes, 74 marathon runners and 74 cyclists after a median of 5.6 [quartiles 4,3, 6,4] years. Median age was 44 [35, 50] years at study enrollment. Spiroergometry did not reveal heart rhythm disturbances or significant ECG changes in the study population. LGE CMR revealed myocardial scar/focal fibrosis in 80 of 293 athletes (27%) including 7 athletes (2%) with ischemic subendocardial LGE of the left ventricle (LV), 16 athletes (6%) with major non-ischemic LGE of the LV and 57 athletes (19%) with minor non-ischemic LGE. During follow-up, two athletes experienced SCD. One marathon runner died during a training run and one cyclist died suddenly at rest. Both athletes had ischemic LGE of the LV. The event rate for SCD was 0.7% in the entire study population and 28% in the 7 athletes with ischemic LGE (*p* < 0.001 compared to athletes without LGE).

**Conclusions:**

Our findings indicate that athletes with ischemic LGE due to unrecognized myocardial infarction are at increased risk for SCD. Our findings highlight the value of LGE CMR to detect occult ischemic scar in asymptomatic apparently healthy athletes, which is of importance, since current guidelines do not recommend to incorporate routine cardiac imaging in pre-participation screening. Athletes with ischemic myocardial scar should at least consider to refrain from high-level exercise as an individual decision.

## Key points

- Athletes with ischemic LGE due to unrecognized myocardial infarction are at increased risk for SCD
- Our findings highlight the value of LGE CMR to detect occult ischemic scar in asymptomatic apparently healthy athletes
- Athletes with ischemic myocardial scar should at least consider to refrain from high-level exercise as an individual decision.

## Introduction

In many cardiac diseases, myocardial scar tissue detected by late gadolinium enhancement (LGE) is a risk factor for cardiac arrhythmia and sudden cardiac death [1]. Previous studies in athletes reported an increased risk for cardiac events in this group of ostensibly healthy subjects [2, 3]. However, the currently available longitudinal studies on this topic included fairly old marathon runners with a mean age of 57 ± 6 years [2] or represent a case–control study in athletes with preexisting ventricular arrhythmia [3]. The purpose of this prospective study was to analyze the prognostic relevance of LGE cardiac magnetic resonance (CMR) in middle-aged endurance athletes without known preexisting cardiac disorders.

## Study Design

The local ethics committee (Ärztekammer Hamburg, PV4764-3108-BO-ff) approved the study, and all subjects gave written informed consent. The study was performed in accordance with the standards of ethics outlined in the Declaration of Helsinki. Three-hundred and twelve athletes apparently healthy athletes were prospectively enrolled. Inclusion criterion was a training for a minimum of 10 h per week and regularly participation in competitions. The included athletes underwent CMR as a part of studies which analyzed the prevalence and pattern of focal myocardial fibrosis in competitive athletes. The results of these studies have been published before [4, 5]. LGE CMR was obtained at baseline in all athletes. Follow-up consisted of a standardized questionnaire and an additional phone call in case of incomplete data. An event was defined as fatal myocardial infarction, ventricular tachycardia, ventricular fibrillation or sudden cardiac death (SCD).

### Study Population

Male and female athletes were contacted through advertisements at local sports clubs. They were included with a self-reported regular training of at least 10 h per week and history of at least one completed competition. Subjects with contraindications for CMR were excluded. All subjects reported no cardiovascular disease and all subjects denied any cardiac or illicit medication intake. Athletes underwent the CMR study before the exercise test, which was performed on the same day. Subjects were instructed to arrive rested with no exercise and no alcohol intake in the preceding 72 h. Any food and caffeine intake were restricted in the preceding 3 h before the CMR, and the exercise test was performed 3 h after the CMR.

### Exercise Testing

Cardiopulmonary exercise testing was performed on the same day after CMR using an eddy current braked cycle ergometer (Ergoselect 100, Ergoline GmbH) to determine maximal oxygen uptake (VO_2max_) and ventilatory threshold. A 12-lead electrocardiogram and heart rate were monitored continuously, and blood pressure was automatically measured every 2 min. The ramp incremental step-exercise test was preceded by a 2-min rest period and unloaded cycling (20 W for 3 min), until a steady state was attained. Depending on the subject’s training history, the ramp was continuously increased by 20–40 W/min to bring the participants to the limit of tolerance within 10–12 min of exercise.

Hypertension at rest was defined by standard criteria with a resting bold pressure ≥ 140/90 mmHg [6]. An exercise-induced hypertension was defined as a systolic blood pressure > 210 mmHg for males and > 190 mmHg for females during spiroergometry [7].

### CMR Protocol

Triathletes and marathon runners underwent ECG gated CMR imaging with a 1.5 T Achieva Scanner (Philips Healthcare) using a 5-element cardiac coil. Cyclers were studied on a 3.0 T Ingenia Scanner (Philips Healthcare) using an anterior/posterior phased-array coil with up to 28 active coils elements. Conventional balanced steady-state free-precession (SSFP) cine imaging in the short axis covering the left ventricle (LV) was obtained for volumetry and LV mass. Additionally, after 10 min of bolus injection of 0.2 mmol/kg gadoterate meglumine (Dotarem, Guerbet), end-diastolic LGE images were acquired with standard phase-sensitive inversion recovery (PSIR) sequences in short-axis orientation and in 2-, 3-, and 4-chamber views matching cine images. Details about the scanning protocol were previously reported [8].

### CMR Data Analysis

Two out of four experienced observers (HR, experience > 4 years; MRS, experience > 8 years; AK, experience > 3 years and JS experience > 4 years in reading CMRs) independently and blindly analyzed each CMR data set using cvi42 software (Circle Cardiovascular Imaging Inc.). CMR parameters were normalized to the subject’s calculated body surface area and are given as the mean of the two investigators’ measurements. LV volumes and LV mass were measured on short-axis cine images as previously described [9]. Focal myocardial fibrosis was identified on short- and long-axis LGE images, and the presence of LGE was classified visually according to established criteria as ischemic LGE, major or minor non-ischemic LGE or absent LGE [10]. Size of LGE was estimated using a threshold method with a cutoff of more than five SDs above normal myocardium [9].

### Statistical Analysis

Statistical analysis was performed using SPSS for MacOS version 29.0 (IBM SPSS Statistics, Armonk, NY) and GraphPad Prism for MacOS, Version 9.1.1 (GraphPad Software Inc.). Values are given as median (first [Q1] and third [Q3] quartiles) for continuous data. Categorical data are given as absolutes numbers with percentages and were compared using a Fisher’s exact or chi-squared test, as appropriate. For continuous baseline variables, a Kruskal–Wallis test was performed on baseline variables to study differences between all groups. As post hoc analysis pairwise comparison between the four LGE groups was performed in case of *p* values < 0.05 using Dunn’s test with Bonferroni correction for multiple testing.

## Results

Complete follow-up was available for 293/312 athletes (94%) including 145 triathletes, 74 marathon runners and 74 cyclists after a median of 5.6 [quartiles 4.3, 6.4] years. Median age was 44 [35, 50] years at study enrollment. Spiroergometry showed no heart rhythm disturbances or significant ECG changes in all subjects. LGE CMR revealed myocardial scar/focal fibrosis in 80 of 293 athletes (27%) including 7 athletes (2%) with ischemic subendocardial LGE of the left ventricle (LV), 16 athletes (6%) with major non-ischemic LGE of the LV and 57 athletes (19%) with minor non-ischemic LGE (Table 1). Athletes with ischemic LGE were informed about presence of such LGE and were referred to their general practitioners for further treatment. Athletes with ischemic and major non-ischemic LGE were older at study inclusion with a median age of 50 [48, 61] and 50 [45, 55] years, respectively, compared to athletes with minor non-ischemic LGE with a median age of 42 [34, 47] years (*p* < 0.05, Table 1). Spiroergometry revealed higher median peak systolic blood pressure with 230 [216, 240] mmHg in athletes with ischemic LGE compared to athletes without LGE with 191 [171, 212] mmHg (*p* < 0.05, Table 1). An exercise-induced hypertension was found more frequently in athletes with major non-ischemic LGE with 50% and in athletes with ischemic LGE with 71% compared to athletes without LGE (*p* < 0.05, Table 1). CMR revealed a higher median LV mass index in athletes with major non-ischemic and ischemic LGE with 90 g/m^2^ and 87 g/m^2^, respectively, compared to athletes without LGE with 74 g/m^2^ (*p* < 0.05) and compared to athletes with minor non-ischemic LGE (*p* < 0.05). LGE size was higher in athletes with major non-ischemic LGE compared to athletes with minor non-ischemic LGE (*p* < 0.05) and in athletes with ischemic LGE compared to athletes with minor non-ischemic LGE (*p* < 0.05). However, no difference in LGE size was found between athletes with major non-ischemic LGE and ischemic LGE (Table 1).

**Table 1:** Baseline characteristics of athletes with no LGE, minor and major non-ischemic LGE and major ischemic LGE

	No LGE (*n *= 213)	Minor non-ischemic LGE (*n *= 57)	Major non-ischemic LGE (*n *= 16)	Ischemic LGE (*n *= 7)	*p-value* Between all groups
*Clinical parameters*					
Age, yrs	44 [35, 49]	42 [34, 47]	50 [45, 55] **a**	50 [48, 61] **b**	** < 0.005**
Weight, kg	75 [65, 81]	75 [65, 81]	75 [65, 81]	86 [74, 88]	0.084
Height, m	1.79 [1.73, 1.85]	1.82 [1.75, 1.85]	1.80 [1.76, 1.81]	1.83 [1.71, 1.86]	0.540
BMI, kg/m^2^	23.0 [21.2, 24.1]	23.2 [21.8, 24.8]	24.5 [22.6, 25.1]	24.9 [22.6, 27.8]	0.051
BSA, m^2^	1.94 [1.78–2.04]	1.95 [1.85–2.05]	1.97 [1.87–2.07]	2.06 [1.94, 2.09]	0.190
Hypertension, *n* (%)	25 (12%)	6 (11%)	5 (31%)	2 (29%)	0.116
*Exercise test*					
Systolic BP at rest, mmHg	121 [114, 131]	125 [119, 130]	126 [115, 141]	133 [124, 141]	0.133
Diastolic BP at rest, mmHg	80 [75, 84]	80 [77, 90]	81 [79, 86]	85 [77, 92]	0.248
Peak systolic BP, mmHg	191 [171, 212]	201 [179, 213]	230 [190, 237]	230 [216, 240] **c**	** < 0.005**
Peak diastolic BP, mmHg	90 [80, 100]	90 [80, 98]	80 [79, 89]	80 [69, 102]	0.204
Exercise-induced hypertension, *n* (%)	47 (22%)	15 (26%)	8 (50%) **d**	5 (71%) **c**	** < 0.01**
Heart rate at rest, bpm	58 [51, 65]	62 [51, 75]	56 [49, 62]	61 [55, 79]	0.196
Peak heart rate, bpm	172 [162, 180]	179 [166, 185]	162 [157, 176] **a**	170 [151, 175]	** < 0.01**
ΔHR rest/peak, bpm	113 [104, 120]	114 [105, 124]	109 [95, 121]	97 [92, 104]	0.065
VO_2max_, ml/kg per min	50 [44, 58]	52 [44, 61]	55 [48, 58]	54 [46, 61]	0.545
Maximal power, W	351 [292, 427]	395 [326, 464]	387 [323, 424]	344 [275, 375]	0.096
*CMR parameters*					
LVEF, %	63 [59, 67]	62 [58, 65]	64 [60, 69]	61 [59, 67]	0.291
LV mass index, g/m^2^	74 [64, 84]	75 [65, 81]	90 [84, 93] **a, d**	87 [81, 97] **b, c**	** < 0.001**
LVEDVi, ml/m^2^	97 [86, 107]	96 [88, 110]	105 [99, 118]	92 [90, 97]	0.051
LVESVi, ml/m^2^	36 [29, 42]	37 [32, 43]	38 [33, 46]	38 [30, 40]	0.490
LGE size, %LV	–	1.1 [0.8, 1.5]	2.0 [0.9, 4.0] **a**	5.1 [3.0, 7.9]** b**	** < 0.001**
LGE mass index, g/m^2^	–	0.7 [0.5, 1.0]	1.4 [0.6, 2.3] **a**	2.4 [1.2, 3.4] **b**	** < 0.001**

Values are median (first [Q1] and third [Q3] quartiles) for continuous data or n (%) for categorical data

*BMI* Body mass index, *BSA* Body surface area, *BP* Blood pressure, *HR* Heart rate, *VO*_*2max*_ Maximal oxygen uptake, *LVEF* Ejection fraction, *LV* Mass index left ventricular mass index, *LVEDVi* Left ventricular end-diastolic volume index, *LVESVi* Left ventricular end-systolic volume index, *LGE* Late gadolinium enhancement

^a^*p* < 0.05 for athletes with major non-ischemic LGE compared to athletes with minor non-ischemic LGE

^b^*p* < 0.05 for athletes with ischemic LGE compared to athletes with minor non-ischemic LGE

^c^*p* < 0.05 for athletes with ischemic LGE compared to athletes with no LGE

^d^*p* < 0.05 for athletes with major non-ischemic LGE compared to athletes with no LGE

### Follow-up Data

Two athletes experienced SCD during follow-up. One marathon runner died during a training run and one cyclist died suddenly at rest. Both athletes had ischemic LGE of the LV myocardium. The age of both athletes was higher with 50 and 61 years compared to the median age of the study population. The older athlete had arterial hypertension, with an increased systolic blood pressure at rest with 149 mmHg, an increased LV mass index of 98 g/m^2^ and an LGE size of 7.9%LV. The other deceased athlete had no risk factors for CAD and a normal LV function, mass and volumes on standard CMR. However, the LGE size was 5.1% LV in this athlete. The event rate for SCD was 0.7% in the entire study population and 28% in the 7 athletes with ischemic LGE (*p* < 0.001 compared to athletes without LGE). None of the athletes with major non-ischemic LGE of the LV or minor non-ischemic LGE had a fatal event during follow-up. Nevertheless, two athletes with a minor non-ischemic LGE experienced a non-fatal event during follow-up. One athlete had a non-fatal myocardial infarction, and another athlete experienced a minor non-disabling ischemic stroke during follow-up.

## Discussion

The current study shows, that athletes with ischemic LGE due to unrecognized myocardial infarction are at increased risk for SCD. Athletes with ischemic LGE were characterized by an increased systolic blood pressure under exercise and an increased LV mass index on CMR compared to athletes without LGE indicating relevant structural alterations of the LV myocardium most likely due to repetitive LV pressure overload during exercise [11]. However, athletes with major non-ischemic LGE were also characterized by a higher number of exercise-induced hypertension and an increased LV mass index similar to that of athletes with ischemic LGE. Therefore, hypertension alone is unlikely the explanation for SCD in athletes with ischemic LGE. Athletes with ischemic LGE had the largest LGE size with 5.1 [3.0, 7.9] %LV compared to athletes with minor non-ischemic LGE with an LGE size of 1.1 [0.8, 1.5] %LV (*p* < 0.05) and compared to athletes with major non-ischemic LGE with 2.0 [0.9, 4.0] %LV. However, the difference in LGE size between athletes with major non-ischemic LGE and athletes with ischemic LGE was not significant, which is most likely related to the small group size of 7 athletes with ischemic LGE. A previous study showed in patients with implantable cardioverter-defibrillator that a scar size > 5%LV was an independent predictor of adverse outcome compared to patients with ≤ 5%LV scar size. Patients with an LV ejection fraction > 30% and scar size > 5%LV had similar risk for death or appropriate implantable cardioverter-defibrillator discharge compared to patients with a LV ejection fraction ≤ 30% [12]. Conversely, those patients with LV ejection fraction ≤ 30% and scaring ≤ 5% had similar risk compared to patients with LV ejection fraction > 30% [12]. Therefore, a scare size > 5%LV seems to be a reasonable cutoff to depict patients at risk for SCD. Interestingly, both deceased athletes had an LGE size > 5%LV identifying them as subjects at increased risk for SCD.

The presence of coronary artery disease (CAD) as indicated by ischemic LGE is certainly the key factor for occurrence of SCD in our athletes. Similar data have been previously observed by Breuckmann et al. [2] who showed that three out of 4 marathon runners with coronary events had ischemic LGE on CMR and only one athlete with a coronary event had no LGE on CMR. Coronary angiography revealed significant CAD in all four runners with a coronary event [2]. Therefore, CAD is obviously the crucial factor for occurrence of coronary events and SCD in older athletes with ischemic LGE on CMR.

## Limitations

The low number of SCD events prevented a more detailed evaluation of other co-factors of SCD in athletes.

## Conclusions

In summary, the current study revealed that the overall event rate for sudden cardiac death was low with 0.7% for all athletes. However, our findings indicate that athletes with ischemic LGE due to unrecognized myocardial infarction are at increased risk for sudden cardiac death. This finding is of importance, since current guidelines do not recommend to incorporate routine cardiac imaging in pre-participation screening among asymptomatic individuals with normal exercise stress test [13]. Our data suggest that athletes with ischemic myocardial scar should at least consider to refrain from high-level exercise. Furthermore, our findings highlight the value of LGE CMR to detect occult ischemic scar in asymptomatic apparently healthy athletes.

## Data Availability

The datasets used and analyzed during the current study are available from the corresponding author on reasonable request.

## Abbreviations

CAD: Coronary artery disease
CMR: Cardiac magnetic resonance
LGE: Late gadolinium enhancement
LV: Left ventricle
SCD: Sudden cardiac death
